# Aβ low threshold mechanoreceptors contribute to sensory abnormalities in fibromyalgia

**DOI:** 10.1093/brain/awaf321

**Published:** 2025-09-03

**Authors:** Mathilde R Israel, Richard Berwick, Nisha Vastani, Qin Zheng, Warren Moore, Margot Maurer, Clive Gentry, Anne Marshall, Haoyue Sun, Harvey Neiland, James P Dunham, Otmane Bouchatta, Katy Plant, Saad S Nagi, Håkan Olausson, Uazman Alam, Xinzhong Dong, Stuart Bevan, Andrew Marshall, Andreas Goebel, David A Andersson

**Affiliations:** Wolfson Sensory, Pain and Regeneration Centre, Institute of Psychiatry, Psychology & Neuroscience, King’s College London, London SE1 1UL, UK; Pain Research Institute, University of Liverpool, Liverpool L9 7AL, UK; Department of Pain Medicine, Walton Centre NHS Foundation Trust, Liverpool L9 7AL, UK; Wolfson Sensory, Pain and Regeneration Centre, Institute of Psychiatry, Psychology & Neuroscience, King’s College London, London SE1 1UL, UK; Department of Anesthesiology and Critical Care Medicine, Johns Hopkins University School of Medicine, Baltimore, MD 21287, USA; Pain Research Institute, University of Liverpool, Liverpool L9 7AL, UK; Department of Pain Medicine, Walton Centre NHS Foundation Trust, Liverpool L9 7AL, UK; Center for Social and Affective Neuroscience, Linköping University, Linköping SE-581 83, Sweden; Wolfson Sensory, Pain and Regeneration Centre, Institute of Psychiatry, Psychology & Neuroscience, King’s College London, London SE1 1UL, UK; Wolfson Sensory, Pain and Regeneration Centre, Institute of Psychiatry, Psychology & Neuroscience, King’s College London, London SE1 1UL, UK; Pain Research Institute, University of Liverpool, Liverpool L9 7AL, UK; Center for Social and Affective Neuroscience, Linköping University, Linköping SE-581 83, Sweden; Wolfson Sensory, Pain and Regeneration Centre, Institute of Psychiatry, Psychology & Neuroscience, King’s College London, London SE1 1UL, UK; Pain Research Institute, University of Liverpool, Liverpool L9 7AL, UK; School of Physiology, Pharmacology and Neuroscience, University of Bristol, Bristol BS8 1QU, UK; Center for Social and Affective Neuroscience, Linköping University, Linköping SE-581 83, Sweden; Pain Research Institute, University of Liverpool, Liverpool L9 7AL, UK; Center for Social and Affective Neuroscience, Linköping University, Linköping SE-581 83, Sweden; Center for Social and Affective Neuroscience, Linköping University, Linköping SE-581 83, Sweden; Pain Research Institute, University of Liverpool, Liverpool L9 7AL, UK; Howard Hughes Medical Institute, Johns Hopkins University School of Medicine, Baltimore, MD 21205, USA; The Solomon H. Snyder Department of Neuroscience, Department of Neuroscience, Johns Hopkins University, School of Medicine, Baltimore, MD 21205, USA; Wolfson Sensory, Pain and Regeneration Centre, Institute of Psychiatry, Psychology & Neuroscience, King’s College London, London SE1 1UL, UK; Pain Research Institute, University of Liverpool, Liverpool L9 7AL, UK; Department of Pain Medicine, Walton Centre NHS Foundation Trust, Liverpool L9 7AL, UK; Center for Social and Affective Neuroscience, Linköping University, Linköping SE-581 83, Sweden; Pain Research Institute, University of Liverpool, Liverpool L9 7AL, UK; Department of Pain Medicine, Walton Centre NHS Foundation Trust, Liverpool L9 7AL, UK; Wolfson Sensory, Pain and Regeneration Centre, Institute of Psychiatry, Psychology & Neuroscience, King’s College London, London SE1 1UL, UK

**Keywords:** fibromyalgia, passive-transfer, Aβ low threshold mechanoreceptor, sensory systems

## Abstract

Fibromyalgia syndrome (FM) is characterized by widespread pain and fatigue. People living with FM also experience tactile allodynia, cold-evoked pain, paraesthesia and dysaesthesia. There is evidence of small fibre neuropathy and hyperexcitability of nociceptors in FM; however, the presence of other sensory abnormalities suggests involvement of large diameter sensory fibres. The passive transfer of FM IgG to mice causes cold and mechanical hyperalgesia associated with changes in A- and C-nociceptor function. However, whether FM IgG also confers sensitivity to light touch and whether large diameter sensory fibres contribute to symptoms evoked by cold is unknown.

Here we demonstrate that the presence of sensory abnormalities such as tingling, correlate with the impact of FM, and that people with FM describe the sensation of cutaneous cooling with neuropathic descriptors such as tingling/pins and needles. We find a causal link between circulating FM IgG and the sensitization of large diameter, Aβ low threshold mechanoreceptors (Aβ-LTMRs) to mechanical and cold stimuli in mice *ex vivo* and *in vivo*. In keeping with our experimental observations, a larger proportion of Aβ-LTMRs respond to cold stimulation in people with FM, but in contrast to our results *ex vivo*, the same fibres display reduced responses to mechanical stimuli.

These results expand the pathophysiological role of IgG in FM and will inform future studies of sensory symptoms and pain in people with FM.

## Teaser

Fibromyalgia is a common pain disorder, predominantly affecting women, marked by pain, fatigue, and sensory abnormalities. Israel *et al.* show that tingling and pins-and-needles sensations in fibromyalgia are linked to antibodies that sensitize large diameter mechanoreceptors to touch and cold.

**See Themistocleous *et al*. (https://doi.org/10.1093/brain/awaf384) for a scientific commentary on this article.**

## Introduction

Fibromyalgia syndrome (FM) is a chronic pain condition with a global prevalence exceeding 2%, and a female predominance.^1^ FM is characterized by widespread pain and fatigue, and those affected live with comorbid and overlapping symptoms and conditions.^2-4^ Amongst the symptoms that people living with FM most frequently describe are distressing sensory allodynia (pain to normally non-painful stimuli), paraesthesia (abnormal sensations whether spontaneous or evoked) and dysaesthesia (as with paraesthesia but described as unpleasant), which occur alongside their unrelenting pain.^5^ Pain and the perception of mechanical and thermal stimuli in FM is a process involving the peripheral and central nervous systems.^6,7^ Sensory neurons in the periphery (e.g. skin) are activated by mechanical, thermal and chemical stimulation of their peripheral terminals. In the mouse, these afferent fibres have been extensively characterized by *in vivo* and in *ex vivo* preparations, which has formed the basis for their functional classification.^8-10^ Studies of human afferents in FM have focused on C fibres, because of their obvious involvement in pain and nociception. Electrophysiological investigations in people with FM revealed sensitization of previously insensitive C-fibre afferents (silent nociceptors) to mechanical stimulation, and importantly, 25% of these fibres also exhibited spontaneous, ongoing activity in FM.^11^ The spontaneous activity in silent nociceptors strongly suggests that these fibres are key to pain in FM. Studies of skin biopsies reliably show that 40%–50% of FM patients have a reduced intra-epidermal nerve fibre density analogous to small fibre neuropathology.^12,13^ Together, these investigations demonstrate that FM is associated with both functional and structural abnormalities in peripheral sensory neurons. Our previous study showed that IgG isolated from people with FM sensitizes mice to mechanical and cold stimuli.^14^ In these passive-transfer experiments, we noted associated changes in the mechanical and thermal sensitivities of skin nociceptors (C and Aδ mechanoreceptors).^14^ In an independent assessment of 184 people with fibromyalgia, 68 patients had serum reactivity against targets in rat dorsal root ganglion and binding to NF200 positive neurons, a marker for large, myelinated afferents, was associated with the presence of paraesthesia in these patients.^15^

In this study, we have examined the impact of FM IgG on other fibre types that may contribute to sensory abnormalities in FM. Prompted by our discussions with people with FM about the range of symptoms which they experience, including touch sensitivity, tactile and thermal dysaesthesia we hypothesized that, in addition to nociceptors, FM IgG also affects large diameter sensory fibres. We explored the prevalence of hypersensitivity to light touch and to temperature in people with FM based on their lived experiences, and their responses to clinical questionnaires and microneurography. We have demonstrated for the first time that mice treated with FM patient IgG are sensitive to innocuous mechanical stimuli. Importantly, this behavioural hyperresponsiveness in FM IgG mice was accompanied by a sensitization of fast conducting Aβ low threshold mechanoreceptor (Aβ-LTMR) afferents. FM IgG administration additionally conferred an aberrant ‘cold’ sensitivity in a subset of Aβ-LTMRs and large diameter sensory neurons. Finally, we translated these findings back to people with FM and found altered firing of Aβ-LTMRs in response to cold and mechanical stimulation. Our results strongly suggest that sensory abnormalities evoked by touch and cold temperature in FM are caused by autoreactive IgG.

## Materials and methods

### Sex as a biological variable

The clinical data presented here are from both male and female participants. However, FM predominantly affects women (4:1). We have previously shown that FM IgG causes a reduction in paw withdrawal threshold to noxious mechanical and cold in both female and male mice.^14^

### Clinical study design and study subjects

Independent cohorts of patients were recruited to two separate studies: a phenotyping study aiming to correlate clinical characteristics with immunological phenotypes (‘APIF’, ISRCTN:18414398) and a phenotyping study aiming to determine the role of small fibre neuropathy in FM pain (‘DEFINE-FMS’, ISRCTN:13134437). Research methodology and some data from the first 51 patients in the ‘APIF’ study (see Supplementary Table 1) and 33 patients in the ‘DEFINE-FMS’ study have been presented previously.^16,17^ Patients and healthy volunteers gave consent as per the Declaration of Helsinki. See Supplementary material, ‘Methods’ section) for details of the clinical experimental design and inclusion criteria.

### Purification of IgG from patient serum or plasma

IgG for individual testing was purified as described previously,^14^ using protein G beads (Sigma-Aldrich). Serum was diluted 1:2 with Hartmann’s solution, passed through a protein G column, and the bound IgG was eluted using 100 mM glycine pH 2.3. The pH was adjusted to 7.4 using 1 M Tris pH 8 and the IgG was dialyzed overnight at 4°C in Hartmann’s using a 10 kDa dialysis membrane (Fisher Scientific). The concentration of IgG present after dialysis was determined using a modified Lowry assay (DC protein assay, BioRad) and adjusted by dilution with Hartmann’s solution or by a concentrating dialysis against a sucrose solution (Sigma-Aldrich). Finally, the IgG solution was sterile filtered using syringe-driven 0.2 µm filter units (Millipore), stored at 4°C and used within 3 months.

### Animals

All animal studies followed general ARRIVE guidelines and were approved by local ethics committees. All behavioural experiments in mice were performed at King’s College London.

Female C56Bl/6J mice (8–14 weeks) were purchased from Envigo and were maintained in continuous temperature-controlled, dark/light cycled (12 h/12 h) cages with access to food and water *ad libitum*. Mice were injected intraperitoneally (i.p.) with healthy control (HC) or FM IgG for 3 days (8 mg, 400 µl). All animals were acclimated to cages and experimental room for >30 min prior to testing. No animals were excluded from analysis. The experimenter was blinded to treatment, and mice were randomized to groups between cages. Randall-Selitto paw-pressure (Analgesy-Meter) and 10°C cold plate (both Ugo Basile) experiments were performed as previously described.^14^ For all low threshold mechanical force assays, the mice were placed in Perspex cages with a metal grid floor. The 0.07 g Von Frey filament was chosen as an innocuous punctate stimulus which in control conditions generates a positive response rate of approximately 1 in every 10 applications. A 0.4 g Von Frey filament was also used in the same manner. The filament was placed on the plantar surface until bent and left for 2–3 s. A positive response was recorded when the animal withdrew, flinched or shook the paw in response to the removal of the hair. The cotton bud was used as previously described.^18^ Finally, a fanned paint brush was used to apply a brief brushstroke (direction proximal to distal) on the glabrous hindpaw. Flicks, flinches, licks, shakes or rapid removal of the paw away from the stimuli was considered a positive response. Any movement such as walking or grooming was deemed inconclusive and a non-response. Each hind-paw was tested three times per stimulus type (Von Frey, cotton swab) with 3–5 min break between each plantar application of stimulus (*n* = 6 total responses).

### *In vivo* calcium imaging

The *in vivo* imaging studies were performed at Johns Hopkins University. *Pirt^+/GCaMP^*^6^ mice were anaesthetized with sodium pentobarbital (i.p., 40–50 mg/kg^−1^). GCaMP6 (excitation/emission: 488/500–549 nm) is a sensitive calcium indicator of calcium concentrations in dorsal root ganglion (DRG) neurons.^19,20^ Surgery and imaging were performed as previously described.^19^ Briefly, after confirming deep anaesthesia (maintained using 1.5%/2% isoflurane), a dorsal laminectomy was performed at the spinal level of L4 to L6 to reveal the underlying L4 ganglion. DRG were viewed using a laser-scanning confocal microscope (Leica), fitted with a macro-based, large-objective, fast electron-multiplying closed-coupled device (EM-CCD) camera. Live images were acquired at four frames/3 s at a subdural depth of 0–70 μm with a 5× , 0.5 NA objective. Baseline calcium levels were recorded for 30 s followed by mechanical stimulation delivered using a brush (glabrous skin only) or rodent pincher analgesia meter (both glabrous and hairy skin) (Bioseb). Mechanical stimulation was applied for 15–20 s. For the cold stimulus protocol, the hind-paw was immersed in a custom chamber to allow for an even flow rate of temperature-controlled water at 10°C. A baseline of 30–60 s was recorded before the cold stimulation, which was maintained for 60 s. The hind-paw stimuli were performed in the same order (brush, pinch, cold), with 15 min between each applied stimulus. Calcium imaging was analysed by a blinded observer.

### *Ex vivo* recordings

These studies were performed at King’s College London as previously described.^21^ mice were killed by cervical dislocation or asphyxiation by rising CO_2_. The skins of both dorsal hind-paws with the saphenous nerve attached were then dissected rapidly post-mortem. The skin was perfused with synthetic interstitial fluid (SIF, 32°C, 95% O_2_ and 5% CO_2_): 108 mM NaCl, 3.5 mM KCl, 0.7 mM MgSO_4_, 26.2 mM NaCO_3_, 1.65 mM NaH_2_PO_4_, 1.53 mM CaCl_2_, 9.6 mM sodium gluconate, 5.55 mM glucose and 7.6 mM sucrose. A and C fibre afferents were distinguished and further classified by action potential shape, conduction velocity, adaption, threshold and response to temperature stimuli. Receptive fields were stimulated electrically using a fine tungsten electrode and conduction velocity calculated from distance/latency. Following this, the mechanical threshold was determined using a custom-built mechanical force transducer (Avere Solutions). Finally, the receptive field was isolated using a small metal ring and paraffin wax, and the skin was perfused with cooled SIF for 60 s to a temperature of ∼5–8°C. All data were acquired using a Cambridge Electronic (CED) 1401 acquisition board and Spike2 (Version 8) software.

### Microneurography

Participants were recruited from Aintree University Hospital (DEFINE study), UK; Linkoping University, Sweden, and Liverpool John Moores University, UK (see Supplementary material, ‘Methods’ section for inclusion criteria details). In total, 29 recordings from Aβ slowly adapting (AβSA) afferents were collected from five male (all healthy control participants, HC) and 15 female participants (seven HC and eight FM) (see Supplementary Tables 3 and 4 and Supplementary Fig. 4). Briefly, the recording protocol proceeded as follows: afferent classification, mechanical threshold, 10 g/128 mN sustained Von Frey force step followed by cutaneous cooling protocols to assess cold sensitivity. Neural activity was sampled at 20 kHz and recorded using the ADInstruments data acquisition system (LabChart software v8.1.24 and PowerLab 16/35 hardware). Thermal stimuli were delivered on the receptive field using a TCS II (QST.Lab) and T08 probe (QST.Lab). The baseline temperature was set to 30°C, target temperature to 0°C, 5°C or 15°C, and ramp rates varied (1°C/s–5°C/s). A thermal response was defined as two or more action potentials fired during the dynamic phase of cooling, or Δ20% mean instantaneous frequency for spontaneously active units.

### Data analysis and statistics

Data are presented as individual data points or as mean ± standard error of the mean, and the number of participants, mice or single units studied is indicated by *n*. Data from behavioural *in vivo* experiments and most *in vivo* and *in vitro* immunofluorescence data were tested statistically by unpaired, two-tailed *t*-test, Mann-Whitney test, two-way repeated measures ANOVA followed by Sidak’s or Dunnett’s *post hoc* test, or one-way ANOVA followed by Tukey’s *post hoc* test. Any difference in the proportion of units responding to cold between groups was examined by Fisher’s exact test. Comparisons with a *P*-value of less than 0.05 were considered statistically different.

## Results

### Description of paraesthesia, dysaesthesia and pain on light touch and cooling in people with FM

**Figure 1:**
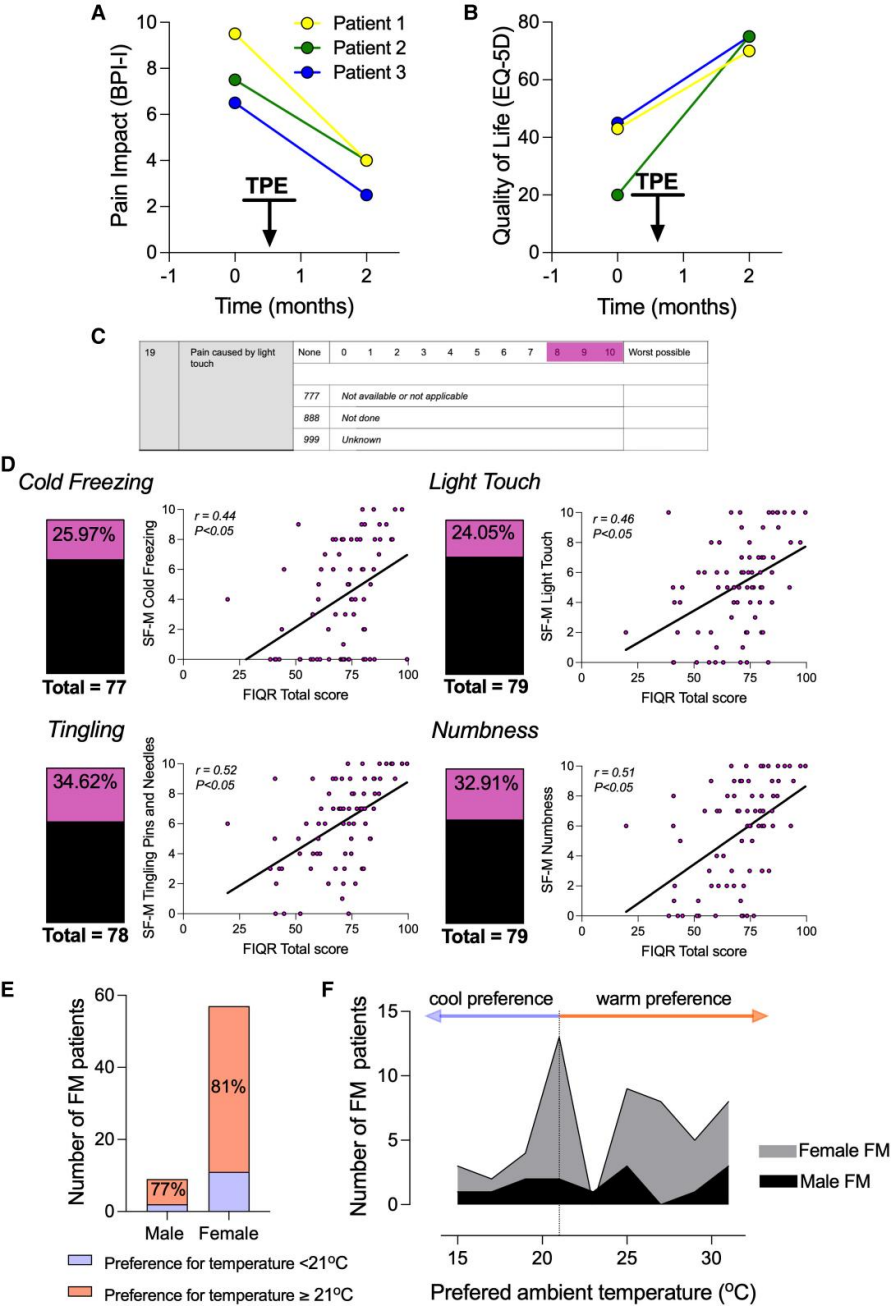
**The presence of self-reported specific neuropathic symptoms (SF-MPQ-2) and ambient temperature preference in a cohort of people with fibromyalgia**. (**A**) Three patients (P1–P3) with severe fibromyalgia (FM) who underwent therapeutic plasma exchange (TPE) described decreased brief pain inventory–impact (BPI-I) scores and (**B**) increased EQ-5D Visual Analogue Scale values post treatment. Data shown at baseline (−1 month prior) and 1 month post TPE. (**C**) Example of SF-MPQ-2 question dark pink indicates high scores (≥8), which are indicated as percentages in **B**. (**D**) Percentage of total cohort with high scores for particular questions in the neuropathic subset of SF-MPQ-2. These scores all have significant moderate (*r* > 0.4) positive correlation with overall scores from the patient’s Fibromyalgia Impact Questionnaire (FIQR). (**E**) In the same cohort, FM patients were asked if ambient air temperature affects their pain (Answer no/yes, female: 12/57 = 69 and male: 1/9 = 10), if yes, they were asked to indicate their preferred ambient temperature. People with FM that affirmed that ambient temperature affected their pain have a preference for warmer ambient temperatures (≥21°C) compared to cooler temperatures (<21°C). Male: 7/9 (77%) and female 46/57 (81%) prefer ≥21°C. (**F**) FM patients show preference for both cool and warm ambient temperatures—data binned per 2°C.

People with FM experience widespread pain and often increased sensitivity to temperature, touch and pressure.^2,22^ Recognized clinical manifestation of large diameter sensory dysfunction includes tingling, dysaesthesias and sensitivity to light touch. In an initial patient and public involvement exercise (PPI), we asked people with FM to describe their sensory abnormalities in more detail. Three patients had previously received therapeutic plasma exchange treatment for their condition, with good subjective outcomes assessed by brief pain inventory—intensity (BPI-I) and the EQ-5D-5L questionnaires (Fig. 1A and B and Supplementary Table 2). Their waste plasma had been retained, and IgG was purified in volumes large enough for preclinical electrophysiological studies (P1 and P2 only). P1–P3 described sensory abnormalities associated with light touch and temperature (see Supplementary Table 2 for patient details and full descriptions), e.g. ‘occasional sensitivity to touch’, light touch feels ‘like sandpaper’, ‘buzzing’ after stroking of skin in cold temperatures, ambient cold increasing the prevalence of other symptoms such as pain and symptom improvement upon warming. We reasoned that qualities such as ‘buzzing’ after stroking of the skin (gentle tactile stimulation) could be mediated by changes in large diameter sensory fibres, as large fibre neuropathy can cause paraesthesia and numbness. Furthermore, such large fibre neuropathy can be caused by autoimmunity, e.g. Sjögren’s syndrome and chronic inflammatory demyelinating polyneuropathy.^23,24^

Therefore, we next examined these phenomena in an established patient cohort (APIF). Other findings from this study are published in Berwick *et al*.^16^ and demographics are presented in Supplementary Table 1. We drew on responses to specific short-form McGill Pain Questionnaire (SF-MPQ-2) questions outlining the quality of experienced pain and related symptoms; these four questions address these specific patient-reported painful and dysaesthetic symptoms (Fig. 1C and D). Each of these four SF-M questions is situated within the neuropathy sub-category of this questionnaire. We analysed the SF-M responses that explore paraesthesia (‘tingling’ and ‘numbness’), mechanical allodynia (‘pain on light touch’) and cold (‘cold freezing pain’). By stratifying the cohort into those who most profoundly experienced these symptoms (scoring ≥8; see Fig. 1C), we found that 24% of patients in the cohort described high intensity ‘pain on light touch’ and ‘cold freezing pain’ in the past week (Fig. 1D). Additionally, more than a third of patients in this group experienced ‘tingling’ and ‘numbness’. Furthermore, within our FM patient cohort, scoring of these four questions was moderately but significantly associated (*r* > 0.4) with the total Fibromyalgia Impact Questionnaire (FIQR) score which denotes overall impact and severity of the condition (Fig. 1D). In contrast, in this cohort, we found no correlation between pressure pain threshold values (PPT), frequently reported in FM, and total FIQR scores (Supplementary Fig. 1).

To assess the relevance of the patients’ description of the impact of cool/cold on their symptoms we asked whether patients prefer a particular ambient temperature. Nine of ten male FM patients (90%) and 57 of 69 female patients (82%) reported that temperature affected their symptoms. When asked to indicate the ambient temperature they preferred, both male and female patients prefer temperatures along a wide spectrum [15°C–31°C (Visual Analogue Scale, VAS)]. However, a great majority of those who had a preference for a particular ambient temperature (>70% males and >80% females) describe a preference for warm temperatures (>21°C) (Fig. 1E and F). We have previously shown that mice treated with FM IgG have increased sensitivity to noxious mechanical and cold stimuli.^14^ Based on the specific patient descriptions above, and the wealth of literature suggesting that patients experience sensory abnormalities alongside pain, we reasoned that Aβ-LTMRs could contribute to the buzzing sensations described by P2 after skin stroking in ambient cold temperatures. Furthermore, we hypothesized that mice injected with FM IgG would also display a heightened sensitivity to innocuous mechanical stimuli like that described by a proportion of people with FM (∼20%–30%; Fig. 1D). Finally, we theorized that any hypersensitivity to innocuous stimuli could be accompanied by sensitization of non-nociceptive skin innervating afferents such as Aβ-LTMRs.

### Passive transfer of IgG causes enhanced nociception and sensitivity to innocuous stimuli *in vivo*

**Figure 2:**
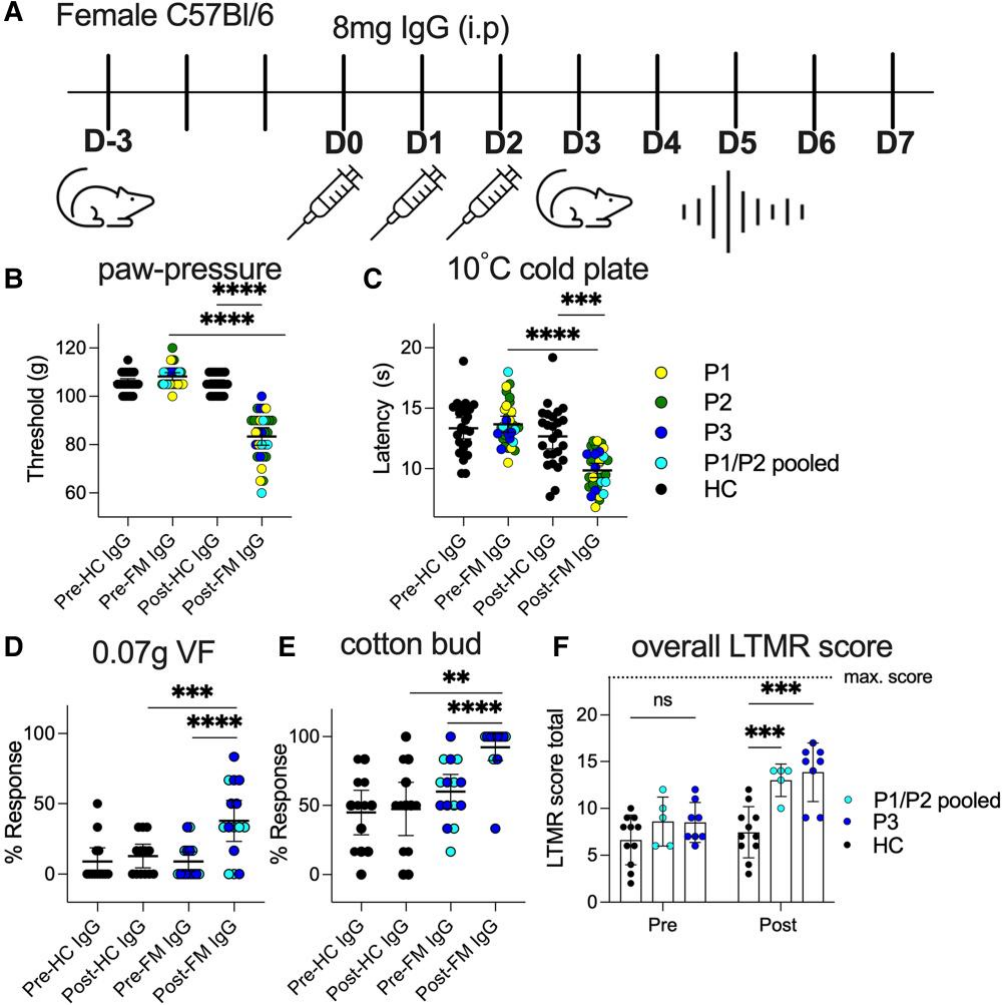
**Administration of fibromyalgia IgG to mice causes hypersensitivity to noxious mechanical and cold stimuli, along with increased responses to innocuous mechanical stimuli**. (**A**) Graphical representation of experimental design. Syringe indicates fibromyalgia (FM)-patient IgG administration [intraperitoneal (i.p.) 8 mg]. Mouse indicates behavioural testing. Signal indicates electrophysiology experiment performed 4–7 days after IgG administration, which coincides with the peak of behavioural response. (**B**) Mice administered either P1–P3 (yellow, green and blue, respectively) individually or P1/P2 combined (turquoise) IgG (i.p. 8 mg) have reduced paw withdrawal thresholds [healthy control (HC): pre 105 ± 1; post 105 ± 1 g versus FM: pre 110 ± 1; post 85 ± 2 g, *P* < 0.05, two-sided paired and unpaired *t*-test, *P* < 0.001 *n* = 25–31]. P1 and P2 injected mice were injected for subsequent use in *ex vivo* experiments. (**C**) Mice administered with either P1–P3 (as for paw pressure) individually or P1/P2 combined had decreased withdrawal latency to the 10°C cold plate (FM: pre IgG 13.7 ± 0.3 versus post IgG 9.9 ± 0.3 s, two-sided paired *t*-test, *P* < 0.0001) compared to healthy control IgG (HC: pre IgG 13.3 ± 0.5 versus post IgG 12.7 ± 0.5 s). (**D**) Mice injected with FM IgG (pooled P1 and P2—teal and P3—blue) had an increased response rate to a low force (0.07 g) Von Frey hair compared to before treatment (9% before and 38% after, *P* < 0.05, two-sided paired *t*-test, *P* < 0 .05, *n* = 15) and between HC- and FM-treated mice (HC post 13%, two-sided unpaired *t*-test, *P* < 0.05, *n* = 13). (**E**) FM IgG administration also significantly increased the response rate to puffed cotton bud on the plantar surface (HC pre 45% and post 47%; FM 60% and post 92%, *P* = 13–15, *P* < 0.05). (**F**) Total score calculated based on responses to four low force stimuli tested 0.07 g, 0.4 g Von Frey hair, puffed cotton bud and fan brush on the glabrous hind-paw. Binary scoring system, 0 no response and 1 response (including rapid lift away from stimulus, flick or shake). The four innocuous stimuli were applied three times to each hind-paw (total, six tests; maximum score possible, 24/24). Mice injected with FM IgG scored higher (post IgG score 13 ± 1and 14 ± 1) than mice injected with HC IgG (post IgG score 7 ± 1).

We performed passive transfer using IgG from patients P1–P3 and P1/P2 pooled 1:1, in accordance with the protocol previously established^14,25^ (Fig. 2A). We confirmed that mice injected with IgG isolated (per individual or pooled) from people with FM displayed hypersensitivity to increasing paw pressure and noxious cold (10°C cold plate) (Fig. 2B and C). In a separate cohort of mice, we tested whether pooled IgG (P1/P2) or IgG from P3 increased the response rate to innocuous mechanical stimulation of the dorsal plantar surface when administered to mice.

We tested a battery of innocuous mechanical stimuli (LTMR selective) on the plantar surface, including 0.07 g, 0.4 g Von Frey filaments, teased cotton-bud and fan paintbrush. The animals injected with FM IgG responded to low force (0.07 g) Von Frey and puffed cotton bud more frequently than mice injected with HC IgG (Fig. 2D and E). Overall LTMR selective scores for mice injected with FM IgG were higher compared to HC IgG injected animals (Fig. 2F; 13–14/24 versus 7/24 post IgG administration).

### Patient IgG sensitizes Aβ low threshold mechanoreceptors to mechanical stimulus *ex vivo*

**Figure 3:**
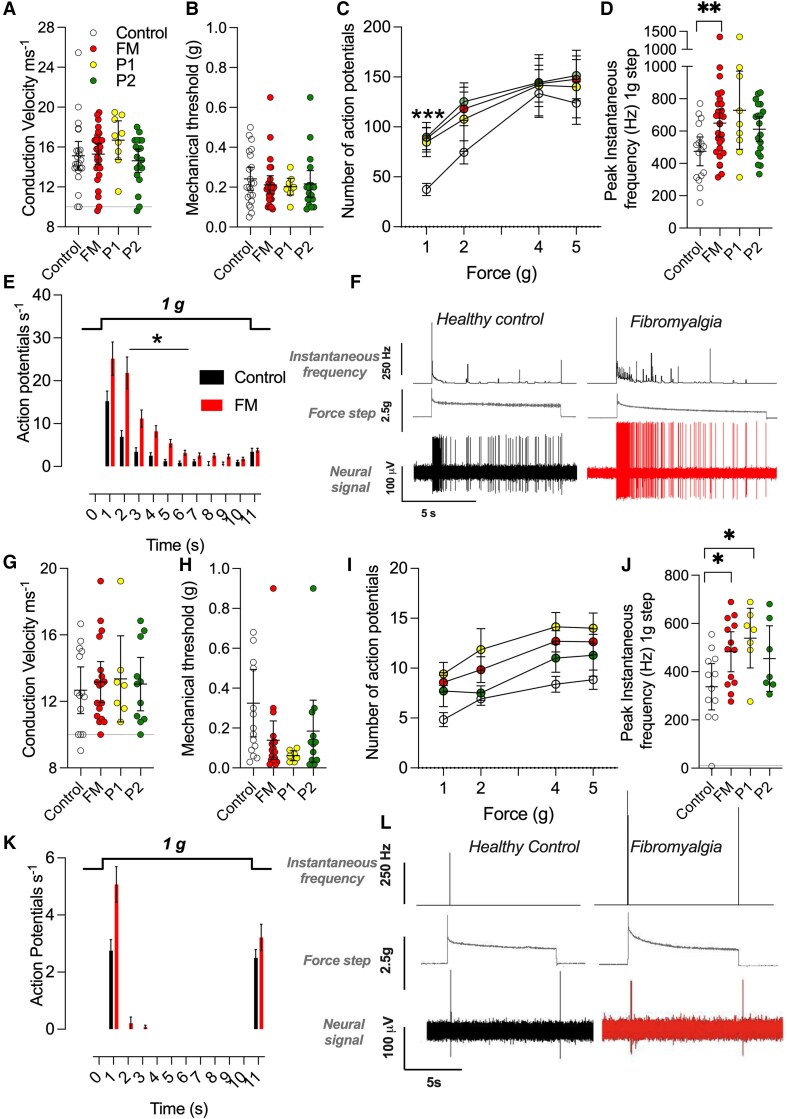
**Fast conducting Aβ-LTMRs *ex vivo* are mechanically sensitized after fibromyalgia IgG administration.** (**A**) Aβ-slowly adapting (AβSA) conduction velocity (line denotes 10 ms^−1^; HC: 15.1 ± 0.8 ms^−1^ FM: 15.3 ± 0.5 ms^−1^) and (**B**) mechanical thresholds (HC 0.2 ± 0.03 g; FM 0.21 ± 0.03 g) remained the same between the treatment groups (total HC: *n* = 23, FM: *n* = 28). (**C**) AβSA from FM IgG-treated animals were sensitized to the lowest mechanical force (1 g) applied firing more action potentials (APs) (HC: 37 ± 6 APs FM: 88 ± 11 APs, HC: *P* < 0.001, two-way ANOVA with Sidak’s multiple comparison, HC: *n* = 18, FM: *n* = 28) at a (**D**) higher peak firing frequency (HC: 474 ± 42 Hz versus FM: 649 ± 42 Hz, *P* < 0.01, unpaired *t*-test, *n* = 18, FM: *n* = 28) than fibres from healthy control treated animals. (**E**) Adaption properties were unchanged between treatment groups with FM treating firing more action potentials throughout (*P* < 0.05 between 2–6 s, two-way ANOVA with Sidak’s multiple comparison). (**F**) Representative traces of AβSA fibres at 1 g force step. (**G**) Aβ-rapidly adapting (AβRA) conduction velocities were not significantly different between treatment groups (HC: 12.7 ± 0.7 ms^−1^ versus FM: 13.2 ± 0.6 ms^−1^; HC: *n* = 14 and FM: *n* = 18). (**H**) Mechanical thresholds of AβRA were lower in skin taken from FM-treated mice (HC: 0.3 ± 0.08 g versus FM: 0.15 ± 0.05 g, *P* < 0.05) in particular P1-treated animals (*P* < 0.001, two-sided unpaired *t*-test, *n* = 7). (**I**) AβRA were sensitized to mechanical stimuli over a range of forces. (**J**) Peak instantaneous frequency of action potential firing significantly higher at 1 g force step (HC: 338 ± 44 Hz versus FM: 483 ± 38 Hz, *P* < 0.05, two-sided unpaired *t*-test, HC: *n* = 12 and FM: *n* = 13). (**K**) Adaption properties were not markedly changed between treatment groups. (**L**) Representative traces of AβRA at 1 g force step. FM = fibromyalgia; HC = healthy control; LTMR = low threshold mechanoreceptors.

Our previous work using *ex vivo* skin saphenous nerve recordings identified that FM IgG administration reduced the mechanical thresholds of mechano-nociceptive afferents (Aδ and C-fibres) in mouse hairy skin.^14^ Here, we examined the hypothesis that low threshold, fast conducting fibres are similarly sensitized to mechanical stimuli by FM IgG administration. Such increased sensitivity of LTMRs including AβSA might be responsible for or contribute to the enhanced sensitivity to low threshold stimuli (cotton bud, Von Frey etc.) observed *in vivo.* However, the mechanical thresholds and conduction velocities of AβSA were unchanged between healthy control and FM IgG-injected animals (Fig. 3A and B). In contrast, AβSA units in preparations from FM IgG-injected mice fired more action potentials (APs) at the lowest force tested (1 g) and correspondingly had higher peak firing frequencies during this force step (Fig. 3C and D). In control conditions,^26^ mouse AβSA fibres sustain an irregular action potential discharge throughout a 1 g 10 s punctate mechanical stimulus applied to the receptive field, the profile of which was unchanged by administration of FM IgG (Fig. 3E and F). However, FM IgG increased the peak discharge rate of AβSA fibres compared to control IgG (Fig. 3D–F).

We similarly tested Aβ rapidly adapting (AβRA) afferents. AβRA fibres, which fire with a characteristic on/off pattern, displayed unchanged conduction velocities (Fig. 3G) but had significantly reduced mechanical thresholds in FM IgG-treated preparations compared to fibres from mice treated with HC IgG (Fig. 3H). This was more apparent in preparations from mice treated with IgG from P1 compared with P2 (Fig. 3H). AβRA from FM IgG-injected mice fired more APs throughout the range of force steps tested (*P* < 0.05 at 1 and 4 g) (Fig. 3I) and exhibited a significantly increased peak firing frequency in response to the lowest force step (1 g) (Fig. 3J). The on/off adaption pattern of AβRA was unchanged between preparations treated with HC or FM IgG (Fig. 3K and L).

### Patient IgG confers an abnormal sensitivity to cooling

**Figure 4:**
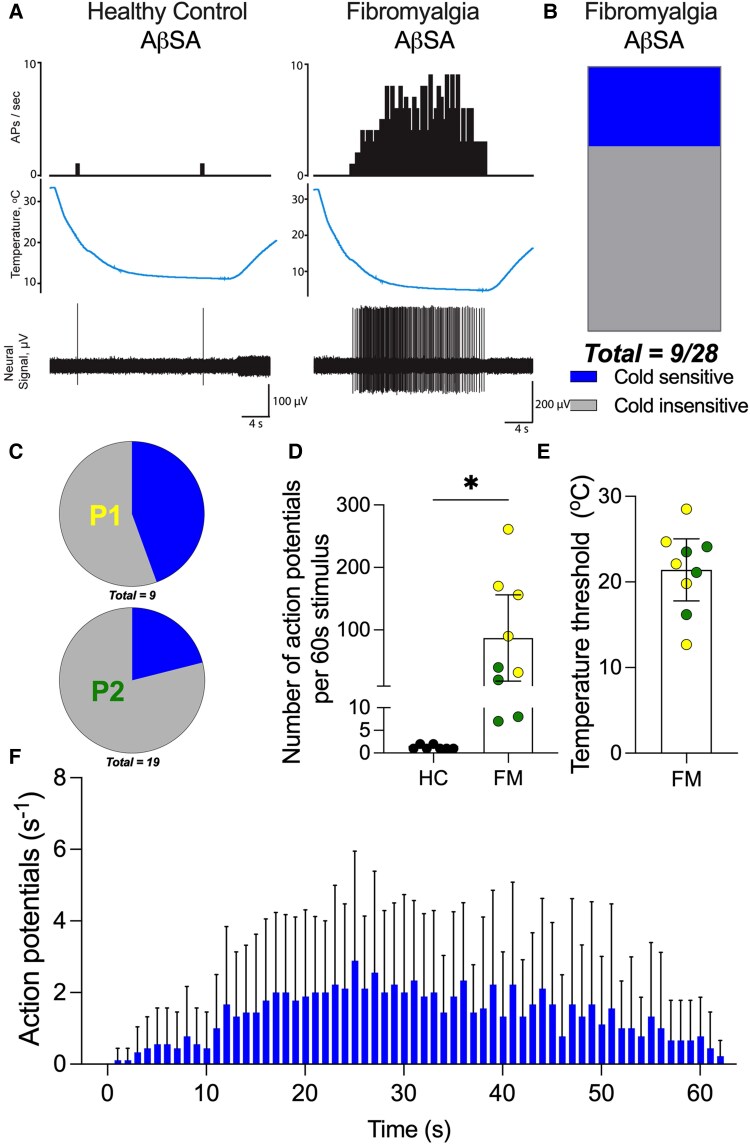
**Aβ-slowly adapting fibres from mice injected with fibromyalgia patient IgG fire to cutaneous cooling *ex vivo.*** (**A**) Representative trace of Aβ-slowly adapting (AβSA) fibres from a healthy control (HC) IgG-treated animal compared to a fibromyalgia (FM) IgG (P1)-treated animal. (**B**) The proportion of fibres (32%) that fired seven or more action potentials during a 60 s cooling ramp (∼32°C to less than 10°C). These cold sensitive AβSA are present in skin taken from mice injected with both patients’ IgG but not HC IgG. (**C**) Pie chart indicates the proportion of AβSA that responded to cold by patient sample administered. P1: cold insensitive, represented in grey (5/9 cold sensitive, blue); and P2: cold insensitive, indicated in black (4/19 cold sensitive, blue). Grey and black are then used to identify which patient the AβSA recording came from in both **D** and **E**. (**D**) Sum of number of action potentials fired by the cold sensitive AβSA fibres (FM IgG-treated 87 ± 0 action potentials/60 s stimulus; HC IgG-treated animals 1 ± 0 *n* = 7–9). Yellow dots represent AβSA recorded from skin of mice injected with P1 and green dots represent fibres from P2 IgG-administered mice. (**E**) Average temperature threshold of activation of AβSA fibres from FM-injected animals is 21.4°C (±1.8°C). (**F**) Average firing rate of AβSA over the course of the cold ramp in skin from FM treated mice (*n* = 9).

We have previously shown that FM IgG can increase the proportion of C-mechano nociceptors (CM) that respond to cold.^14^ However, the reports of mixed temperature/tactile sensitivity, particularly those given by P1 (pain due to holding a cold element, e.g. a fork) and P2 (‘buzzing’ after stroking in the cold), led us to explore any contribution of Aβ-LTMR to aberrant cold sensing. Therefore, we also challenged the Aβ afferents described above with 60 s temperature ramps from 32°C to less than 10°C. In both naïve (not shown) and HC-treated preparations, we observed some AβSA (HC: 7/23) units that discharged up to three APs during cooling ramps (Fig. 4A). Firing to cooling has been reported previously in this class of fibres in mammals.^27-31^ However, in FM IgG-treated animals, about 30% of AβSA (9/28) fired seven or more APs during the cooling phase of the cold ramp and were therefore classified as ‘cold sensitive’ (Fig. 4B and C). We could not identify any unit displaying the same level of activity in preparations from HC-treated animals (average 1 ± 1 AP/per 60 s stimulus). The firing rate of these FM AβSA was heterogeneous, ranging from 7 to 261 APs (average number of APs 87 ± 30) (Fig. 4D). While cold-responsive AβSA were identified in preparations from mice treated with either of the two patient donors, AβSA units with a high frequency (>100 AP/60 s cold ramp) of cold evoked AP discharge (e.g. Fig. 4A and D) were found exclusively in mice injected with IgG from P1.

The temperature threshold of activation, defined by the temperature at which the second AP was discharged for FM IgG-treated mice was on average 21.4^o^C (±1.5^o^C) and could not be reliably obtained for HC AβSA, which only fired 1–2 APs (Fig. 4A and E). The conduction velocity of cold sensitive AβSA (15.8 ms^−1^ ± 1, *n* = 9) was not significantly different from either HC (15.2 ms^−1^ ± 0.7, *n* = 19) or non-cold sensitive AβSA from FM IgG-injected animals (14.8 ms^−1^± 0.6, *n* = 19).

### Calcium influx in larger diameter DRG neurons evoked by plantar cold stimuli in mice injected with FM IgG

**Figure 5:**
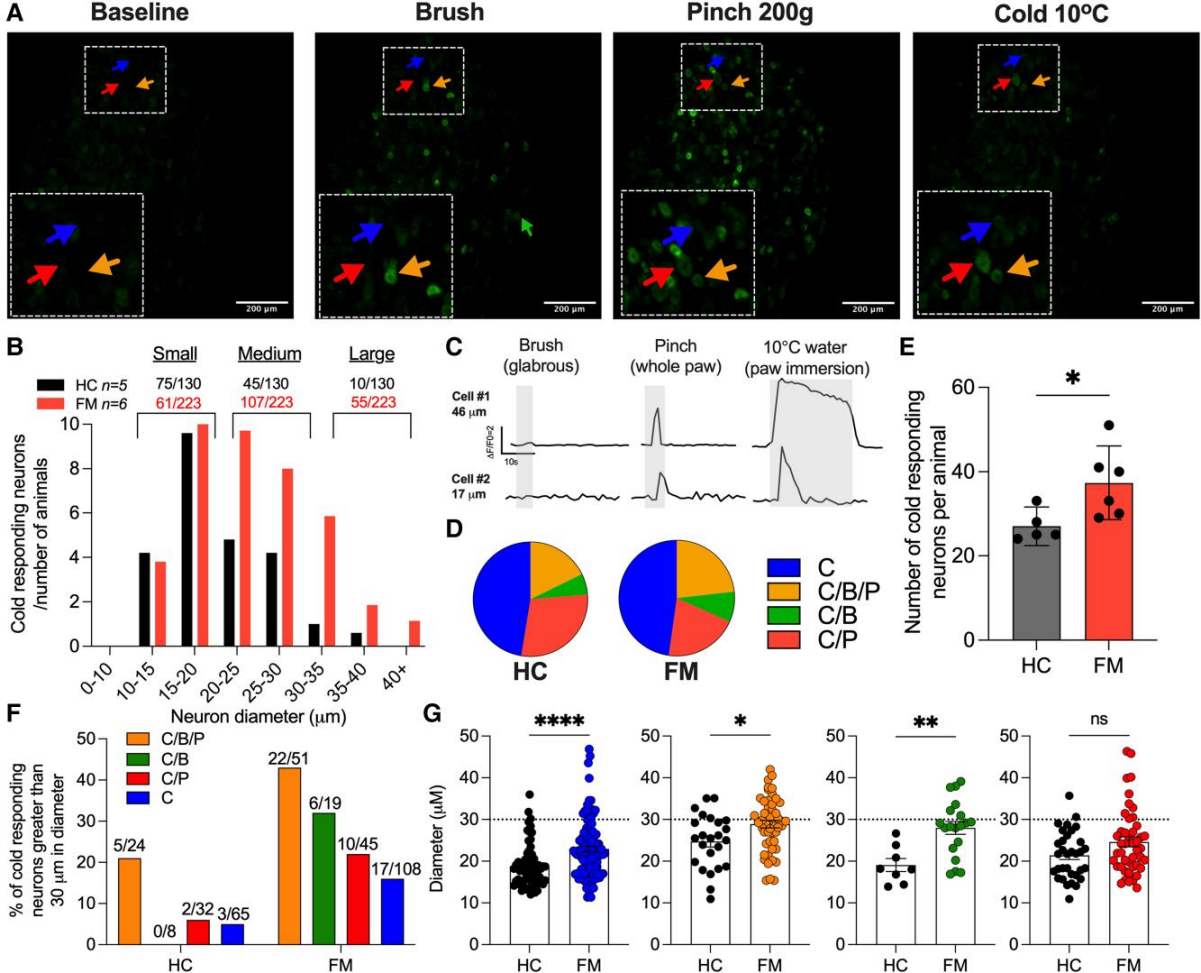
**Mice injected with IgG from people with fibromyalgia have more large diameter dorsal root ganglion neurons responding to cold *in vivo.*** (**A**) Representative image of *in vivo* calcium imaging of dorsal root ganglia (DRG) of mice injected with fibromyalgia (FM) IgG demonstrates calcium influx in larger diameter fibres after submersion of hind-paw in a 10°C water bath, brush and pinch stimuli. Coloured arrows indicate DRG that responded to cold alone (blue), cold and pinch (red), cold and brush (green) and cold, brush and pinch (orange). (**B**) In healthy control (HC) IgG-treated animals, the majority of DRG neurons that responded to cold temperature with calcium influx were small in diameter. In FM IgG-injected animals, there was an increase in the number of large diameter (>30 µm) DRG neurons responding to cold. (**C**) Trace of calcium influx during paw immersion in 10°C water in a large diameter (>40 µm) and small diameter (17 µm) neuron. (**D**) Overall polymodality of all the cold responding neurons remained similar between HC and FM. (**E**) The average total number of cold responding DRG neurons per animal was increased in mice treated with FM IgG (HC 27 ± 2 and FM 37 ± 3 neurons, *P* < 0.05, two-sided unpaired *t*-test, *n* = 5 HC and FM *n* = 6 animals). (**F**) The number and percentage of cold responding neurons that were greater than 30 µm in diameter of each class; cold alone (blue), cold and pinch (red), cold and brush (green) and cold, brush and pinch (orange). (**G**) A comparison of the diameter of fibres that responded to cold alone or cold and other mechanical stimuli demonstrated that large diameter fibres (line denotes 30 µm) responded to cold across all groups. In HC IgG-treated animals, fewer medium to large diameter neurons responded to brush and cold stimuli (two-sided unpaired *t*-test, *P* < 0.01).

We next asked whether these cold sensitive Aβ units are also present in intact, alive mice after FM IgG treatment. We therefore turned to *in vivo* Ca^2+^-imaging of the L4 (hind-paw innervating afferents) DRG (expressing GCaMP6) of anaesthetized mice treated with FM or HC IgG. This technique enabled the investigation of sensory nerve activity *in vivo* in response to a range of stimuli, including cold, brush and pinch (Fig. 5A). Consistent with the increased cold responsiveness of Aβ (Fig. 4) and C-fibres^14^ in preparations from FM IgG-treated mice, we observed that cooling of the paw evoked [Ca^2+^]_i_-responses in larger diameter DRG neurons in mice treated with IgG from P1 and P2, compared to HC IgG (Fig. 5A–C). The average diameter of responding neurons in FM IgG-injected mice was increased significantly compared to HC mice, consistent with our electrophysiological identification of Aβ fibres with abnormal cold responsiveness (Fig. 5B and C). Cold responding neurons responded to other mechanical modalities including pinch and brush in similar proportions (Fig. 5D). Overall, significantly more neurons responded to cold in FM IgG- compared to HC IgG-treated animals (Fig. 5E). However, the number of small neurons (with a diameter of 10–20 µm) that responded to cold with Ca^2+^-influx remained similar in HC and FM IgG-treated animals (Fig. 5A and B). Very few large diameter neurons (>30 µm) respond to cold in mice administered HC IgG (eight neurons, five mice), but this number was increased significantly after FM IgG treatment (61 neurons, six mice) (Fig. 5B). The large diameter (>30 µm) neurons that responded to cold in FM-treated mice also responded to pinch and brush (Fig. 5F).

Thus, FM IgG specifically conferred cold sensitivity to larger diameter, A-fibre forming neurons. Similarly, in animals injected with FM IgG, the diameter of the DRG neurons that responded to both brush (LTMR activation) and cold was larger than in HC IgG-treated mice (Fig. 5F and G). Polymodal (cold, brush and pinch sensitive) neurons in FM IgG-treated mice were also larger in size compared with those administered with HC IgG (Fig. 5G).

### People with FM describe cold pain differently from healthy control participants

**Figure 6:**
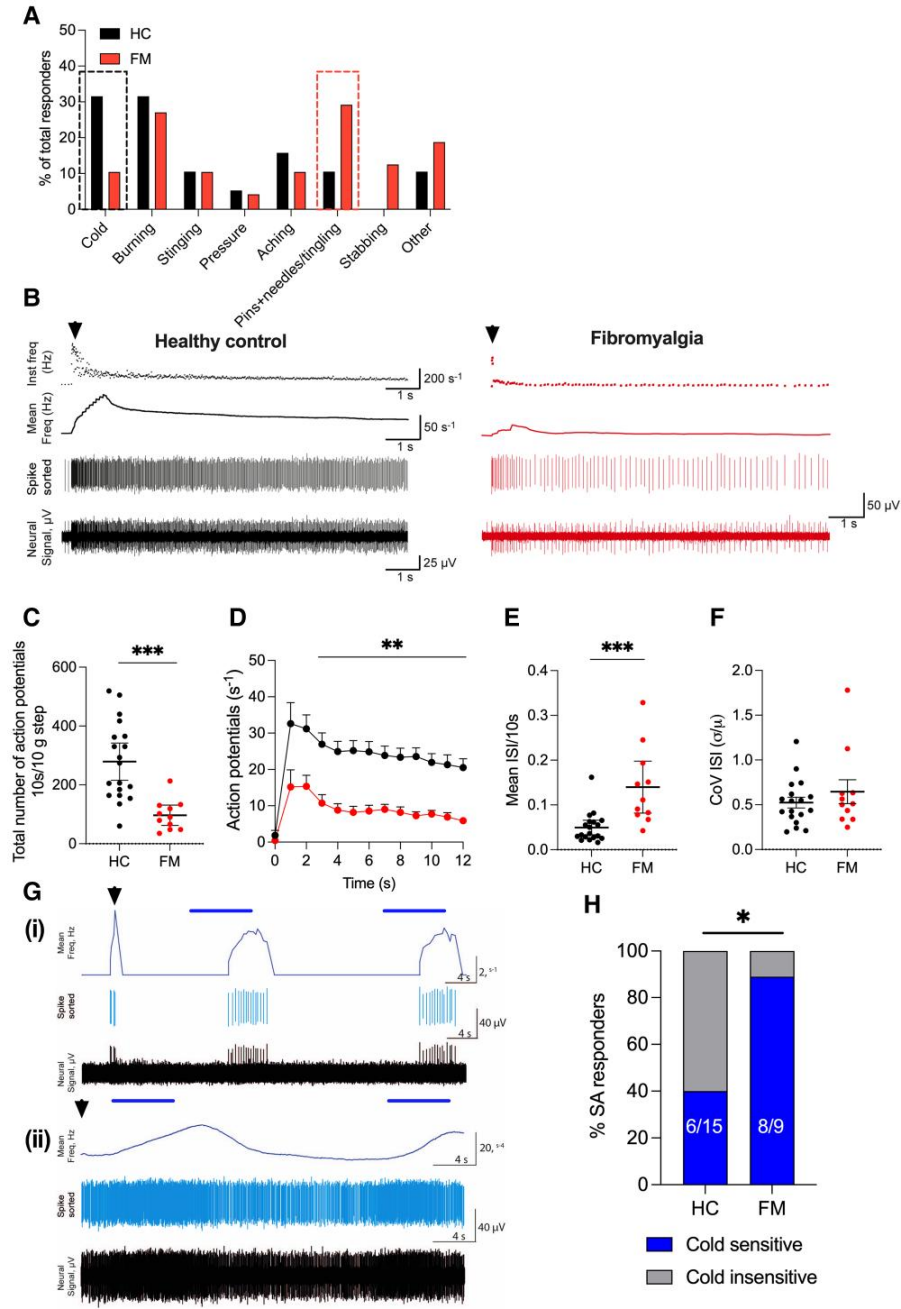
**People with fibromyalgia have an altered perception of cold stimuli alongside changes to the firing of Aβ-slowly adapting fibres.** (**A**) People living with fibromyalgia (FM) were asked to describe the sensation of cooling during Quantitative Sensory Testing (QST) use different phrases compared to healthy control (HC) participants. Dashed line highlights the reduction in the number of patients saying that the cold stimulus was cold (HC: 6/22 and FM 5/49) and the concurrent increase in number of people who describe the sensation as like ‘pins and needles/tingling’ (HC 2/22 and FM 14/49). (**B**) Compared to Aβ-slowly adapting (AβSA) afferents recorded from HC (*left*, black), AβSA in people with FM (*right*, red) fire fewer action potentials during mechanical indentation of receptive field patients with Von Frey hair. (**C**) Average number of action potentials fired by AβSA in the first 10 s of the 10–13 g Von Frey stimulation was reduced in FM (97 ± 15 AP, *n* = 12) compared to HC participants (286 ± 31, *n* = 18, two-tailed unpaired *t*-test, *P* < 0.05). See Supplementary Fig. 2 for a breakdown of response by sex and by site. (**D**) The number of action potentials binned per second demonstrates that the adaption pattern remained similar between FM and HC AβSA afferents, with a lower number of action potentials across both the dynamic and static phases of indentation. (**E**) The mean inter-spike interval (ISI) was increased in FM patients (HC 0.05 ± 0.01 s *n* = 18: FM 0.14 ± 0.03 s *n* = 11, two-tailed unpaired *t*-test, *P* < 0.05) without affecting the (**F**) coefficient of variance (CoV) of ISI indicated that both FM and HC AβSA fired regularly to mechanical stimuli (HC 0.53 ± 0.27 *n* = 18: FM 0.65 ± 0.44 *n* = 11). (**G**) Representative recordings of two cold sensitive AβSA afferents: (**i**) an AβSA afferent that did not continuously fire in response to the thermode being placed (indicated by the black arrow) on the receptive field compared to (**ii**) an AβSA afferent which fired continuously after the thermode was positioned on the receptive field. The time of the dynamic phase of the cooling ramp is shown as the blue line above the trace. In the *bottom* trace, a clear increase in firing (>Δ20%) was observed during the dynamic phase of cooling. (**H**) The proportion of cold sensitive AβSA was increased in FM, represented as a percentage (HC 6/15 versus FM 8/9, two-tailed Fisher’s exact test, *P* < 0.05).

The patients’ reports of their experience of pain and paraesthesias/dysaesthesias in cold ambient temperatures and touching cold objects, coupled with the activation of large diameter Aβ fibres by cold ramps in preparations from mice injected with FM derived IgG, prompted us to ask how people with FM described the sensory percepts of cooling or cold and whether this was different to HC participants. Therefore, arising from our preclinical findings, we adapted the standard Quantitative Sensory Testing (QST) protocol to include a description of sensations that arose during cold pain testing. Individuals participating in the DEFINE-FMS clinical study (DEFINE, see ‘Materials and methods’ section) were asked to describe the sensation at cold pain threshold. No verbal prompts were given by the tester before each participant’s response was recorded. We found that 30% of people with FM (14/49) describe the sensation of cooling during these tests with the phrases ‘pins and needles’ or ‘tingling’ compared to 10% (2/22) of healthy participants. Furthermore, only 10% of participants with FM (5/49) use the phrase ‘cold’ to describe the stimulus compared to 30% (6/22) of healthy participants (Fig. 6A). People were also asked to describe the sensation during the heat testing protocols and overwhelming the descriptor used was ‘heat’ with only one FM patient reporting ‘pins and needles’ during heat testing.

Mechanically sensitive AβSA afferents respond to cutaneous cooling in humans,^28,29^ and we therefore asked if the enhanced sensitivity observed in AβSA fibres from mice injected with patient IgG could also be detected in FM patients using microneurography. Unexpectedly, unlike in mice injected with patient IgG, AβSA afferents in people with FM fire significantly fewer APs in response to suprathreshold Von Frey stimuli compared to healthy participants (Fig. 6B–D). The mechanical thresholds of AβSA were equivalent in HC and FM (HC: 0.15 ± 0.02 grams force, *n* = 10 versus FM: 0.15 ± 0.02 grams force, *n* = 16). Correspondingly, the average inter-spike interval (ISI) of AβSA increased during mechanical indentation in people with FM (Fig. 6E). However, the coefficient of variance (CoV) of the ISI was unchanged between HC and FM AβSA, indicating a similar pattern of firing (Fig. 6F). Finally, a thermode was placed on the AβSA receptive field during microneurography, and cooling ramps were applied to assess cutaneous cold sensitivity of the afferent. Due to their low threshold mechano-sensitivity, 7/21 AβSA (from both HC and FM) fired continuously following application of the thermode to the skin. These afferents typically display reduced firing rate during heating and increased firing upon a fall in skin temperature (see Konietzny^31^ and Supplementary Fig. 2). Therefore, AβSA were classified as cold sensitive if they either fired two or more APs during dynamic cooling [Fig. 6G(i)] or, in the case of ongoing activity, had a greater than 20% increase in the rate of firing during the dynamic phase of cooling [Fig. 6G(ii)]. In HC, 6 of 15 (40%) AβSA were cold sensitive compared to 8 of 9 in FM (88%, *P* < 0.05 Fisher’s exact test) (Fig. 6H).

## Discussion

Pressure sensitivity is an established hallmark of fibromyalgia, but other sensory abnormalities also feature prominently.^2,32^ Sensory symptoms other than pain, such as dysaesthesia and sensitivity to ambient temperature, are an often overlooked and important burden for those living with FM. Here, we provide evidence that autoantibodies contribute to the peripheral neurobiological basis of these symptoms. We confirm earlier observations of an increased sensitivity to cold in people with FM compared to HC and describe that FM participants use a broader range of perceptive terms to describe the sensory quality associated with cold.^22^ We have previously demonstrated that administration of IgG from people living with FM to mice transfers hypersensitivity to noxious mechanical and cold stimuli, and here we show that autoantibodies might also be responsible for sensory abnormalities produced by innocuous tactile or cold stimulation of fast-conducting LTMRs. We show for the first time that IgG isolated from people with FM, who specifically described tactile and thermal sensory abnormalities, is sufficient to cause sensitivity to light touch in mice. Furthermore, cutaneous Aβ-LTMR in FM IgG injected mice were sensitized to both mechanical and cold stimulation. This finding was further validated *in vivo*, by observing [Ca^2+^]_i_-responses in large diameter DRG neurons during cooling of the afferent terminals in the skin in mice treated with FM IgG. Our *ex vivo* studies of skin-nerve preparations from mice injected with human IgG strongly indicate that aberrant cold sensitivity of large diameter, fast-conducting AβSA fibres might at least in part be responsible for the cold and touch evoked sensitivity and paraesthesias/dysaesthesias reported by people with FM.^22^ Finally, we translate our bench-side experimental findings back into humans by using questionnaires and microneurography to assess modality specific activity of AβSA afferents. AβSA fibres in people living with FM displayed reduced responses to the mechanical stimulation, but an increased proportion of these fibres responded to cooling.

The therapeutic plasma exchange treatment reported in this study serves as a proof-of-concept about the safety and feasibility of this treatment delivery in people with FM while simultaneously delivering large volumes of plasma for IgG isolation and preclinical studies. Future double-blind, placebo-controlled trials should follow to assess side-effects and unspecific treatment effects in the control arm. Any feasibility study should include a sham group, additional data points and objective measures such as quantitative sensory testing and intra-epidermal nerve fibre density measurements. Touch evoked sensitivity, such as that described by P1–P3 in this study, has previously been reported as a clinically relevant complaint in about 20% of people with FM, whereas 24% reported significant thermal hypersensitivities.^33,34^ These incidences agree well with our findings using the subset of questions focused on neuropathic pain in the short-form McGill pain questionnaire. There are abundant QST studies in FM,^4^ sometimes with conflicting results. Several studies report that people with FM display hypersensitivity to cold in QST,^2,35,36^ along with loss-of-function in parameters that are likely to be associated with modulation of LTMR function (e.g. vibration sensitivity).^35,37-39^

Here we have shown that IgG from FM patients sensitizes low threshold sensory afferents in mice. Our previous passive-transfer experiments demonstrated an enhanced sensitivity in mechano-nociceptive afferents, including reductions in mechanical thresholds (Aδ and C-mechano receptors) and increased responsiveness to noxious cold (in C fibres).^14^ In passive-transfer studies with IgG from people with complex regional pain syndrome (CRPS), we noted sensitization of nociceptive A and C fibres, but low-threshold Aβ and Aδ units did not fire more APs to increasing mechanical force steps.^40^ Unlike FM, CRPS is typically a post-traumatic condition, and in these earlier studies,^40,41^ IgG was delivered in combination with a paw incision as a minor experimental insult. This provides the opportunity to examine the direct effects of CRPS IgG in the uninjured paw, as well as in the post-surgical environment of the injured paw. CRPS IgG exacerbated and prolonged the sensitivity to noxious mechanical stimuli in the injured paw but left the uninjured paw unaffected.^40^ Importantly, and unlike FM-IgG, CRPS-IgG did not affect tactile sensitivity in either paw.^40^ Furthermore, the mechanical hypersensitivity produced by transfer of CASPR2 autoantibodies from patients with neuropathic pain to mice was not accompanied by Aβ fibre abnormalities but instead by increased activity of Aδ-LTMRs.^42^ The results presented here on the sensitivity to innocuous mechanical stimulation *in vivo*, and of Aβ-LTMR function in *ex vivo* preparations, thus appear specific to FM IgG, since they were not observed with IgG from pain-free volunteers or from patients with other painful disorders where autoreactive IgG is present. While administration of patient IgG to mice transfers sensory symptoms associated with CRPS, FMS, rheumatoid arthritis or CASPR2 autoantibodies,^42-44^ the phenotypes produced are specific to each condition and not simply indicative of pain or autoreactive IgG.

We have not assessed cold sensitivity of Aδ-fibres in people with FM using microneurography. Putative Aδ nociceptor involvement warrants further investigation as Aδ afferents have an established role in cold sensation,^45^ and the amplitude of Aδ-specific pain-related evoked potentials is reduced in people with FM, albeit via unknown mechanisms.^13^ We cannot yet explain the cellular mechanisms through which patient-derived IgG affects multiple classes of mouse sensory afferents in different ways. However, we expect that the combined effect on all afferents contributes to the increased sensitivity to temperature, touch and paraesthesia observed in people with FM. Previous studies show that FM IgG display an increased reactivity to satellite glial cells in the DRG, and this binding appears related to symptom severity.^25,46,47^ However, this cellular reactivity is also found in a subset of pain-free healthy volunteers,^47^ and it remains to be clarified whether this interaction is causally involved in the pathophysiology of FM, and if a single antigen is responsible. Intriguingly, the extent of binding of FM sera to NF200 positive, large diameter myelinated neurons in rat DRG sections has been reported to be associated with the presence of paraesthesia in these patients.^15^ One possible explanation for the observed hypersensitivity of several different fibre classes is the presence of multiple antigenic targets for the patient IgG. It is also possible that different transduction mechanisms and the repertoire of ion channels expressed by sensory afferents may explain why FM IgG produce subtly different abnormalities in each class of sensory afferent. Furthermore, the results of this study do not preclude the involvement of other immune cells and mediators in the development of FM symptoms.^48,49^ Such dysregulation of other mediators and immune cells may on the one hand be required for the generation of pathological autoantibodies but may on the other also be involved in the downstream effector mechanisms of FM IgG.

This work adds to the small number of studies that have described changes in large diameter fibres in FM.^39,50-53^ Microneurography studies of afferents in people with FM have demonstrated *de novo* mechanical sensitivity of some mechanically insensitive C fibres, as well as spontaneous C fibre activity.^11,35^ However, this study is the first to show both gain- and loss-of-function of Aβ-LTMR in people with FM. Although understudied in the context of painful disorders, AβSA fibres are sensitive to temperature changes in humans and non-humans.^28,29,31^ The precise ionic mechanism and any biological relevance of cold transduction in these afferents is unknown. However, RNA sequencing analysis of human and mouse sensory neurons suggests that it is unlikely through canonical and putative cold transduction channels such as TRPM8, TRPA1 or Gluk2, which are essentially absent.^54-56^ Furthermore, the increased response of AβSA fibres to cold demonstrated in this study is unlikely to result in the perception of cold-evoked pain or allodynia. Our previous work demonstrated that FM IgG is sufficient to cause increased sensitivity to cold in C nociceptors in mice^14^; however, we are not aware of any microneurography studies that explore whether C-nociceptors respond more to cutaneous cooling in people with FM. AβSA fibres are crucial for tactile discrimination^57^ and, therefore, the reduction in mechanical sensitivity of these afferents in FM may contribute to the clumsiness described by some patients.^58^ Results of sensory nerve conduction studies in people with FM compared to healthy controls are inconclusive, however, a decrease in sensory nerve action potential amplitude has been reported previously.^59,60^ Furthermore, though likely through different ionic mechanisms to FM IgG-evoked sensitivity, patients with large diameters sensory nerve dysfunction due to chemotherapeutic agents (e.g. platinum-based drugs) also report paraesthesia and cold evoked pain.^61^

We cannot provide a mechanism detailing the changes that occur in patients’ AβSA fibres that result in reduced firing to supra-threshold mechanical stimuli. We observed changes in the mouse behaviour and skin afferents within days after administration of IgG; however, some of the findings were in the opposite direction to those found in human AβSAs. In patients, the road from symptom onset to diagnosis is long, averaging greater than 6 years.^62^ In contrast, animals were only administered patient IgG for three consecutive days. Therefore, repeated or ongoing IgG treatment should be explored in future studies. Additionally, we cannot rule out that this discrepancy between human and mouse data is due to immunological or neuronal differences between species. Nevertheless, our pre-clinical electrophysiological experiments in rodents identified a class of afferents previously unexplored in FM pathophysiology, and our studies of human subjects demonstrate that these fibres are also dysfunctional in people with FM. Whether these functional changes are responsible for clinical symptoms remains to be elucidated. However, the presence of super-fast conducting A-nociceptors, a proportion of which are cold sensitive, challenges the dogma that nociceptive signals are carried solely or predominantly by slower C-fibres.^63,64^ The positive association between ‘numbness’ and ‘tingling’ and fibromyalgia impact (FIQR) scores suggests that paraesthesia and light touch sensitivities may be directly related to the daily impact of FM symptoms in people. The increased use of such terms to describe the sensation of cutaneous cooling, which we show activates a larger proportion of Aβ-LTMRs in FM, provides a link between temperature-evoked dysesthesia, large diameter dysfunction and symptom impact. Furthermore, the compromised function of Aβ-LTMRs produced by administration of FM IgG provides new insights into the possible neurobiological processes that produce symptoms. Our findings further emphasize the importance of peripheral sensory afferents as the origin of pain and sensory abnormalities in FM.

## Supplementary Material

awaf321_Supplementary_Data

## Data Availability

The data that support the findings of this study are available on request from the corresponding authors. Some of the data are not publicly available due to their containing information that could compromise the privacy of research participants.
