# Supplementary material for: Aβ low threshold mechanoreceptors contribute to sensory abnormalities in fibromyalgia
Source: Brain. 2025 Sep 3;148(11):4016–29. doi: 10.1093/brain/awaf321 (PMC12588719; doi:10.1093/brain/awaf321)
Supplement: awaf321_Supplementary_Data [file awaf321_supplementary_data.zip › brain-2025-00414-File008.pdf]

Supplementary Table 1: APIF patient characteristics

Characteristics		Male	Female	*P*
**Demographics**				
	n	15	64	
	Age (yrs.)	42.0 (33.0-48.0)	52.0 (49.0-55.0)	*0.0018*
	BMI (kg/m^2)	31.5 (28.3-45.4)	33.7 (31.0-36.9)	*ns*
	Children	53.8.0% (7/13)	74.6% 44/59	*ns*
	Years of Diagnosis	4.9 (2.6-12.5)	5.4 (4.3-8.0)	*ns*
	Years of WSP Symptoms	9.3 (5.5-15.4)	10.3 (8.0-13.1)	*ns*
**Impact Indices**				
	BPI (0-10)	8.0 (5.4-8.4)	7.7 (7.1-8.1)	*ns*
	FIQR (0-100)	67.3 (41.1-80.7)	73.6 (70.0-76.5)	*ns*
	EQ5D Health (0-100)	50.0 (20.0-65.0)	45.0 (40.0-50.0)	*ns*
	Lack of Energy (0-10)	8.0 (7.0-9.0)	9.0 (8.0-9.0)	*0.0431*
	HADSA (0-22)	12.0 (10.0-16.0)	12.0 (11.0-13.0)	*ns*
	HADSD (0-22)	12.0 (9.0-15.0)	12.0 (10.0-13.0)	*ns*
	PCS (0-52)	25.0 (18.0-44.0)	23.5 (19.0-28.0)	*ns*
	SSS (0-12)	10.0 (9.0-11.0)	10.0 (10.0-11.0)	*ns*
	WPI (0-19)	14.0 (12.0-15.0)	15.0 (14.0-16.0)	*ns*
	TPs	12.0 (10.0-17.0)	16.5 (15.0-18.0)	*0.0139*
	PSEQ	19.0 (10.0-29.0)	20.0 (17.0-23.0)	*ns*
**Pain Indices**				
	Pain Now (0-10)	6.5 (4.0-7.0)	7.0 (7.0-8.0)	*0.0044*
	Pain Worst 24hrs (0-10)	7.5 (6.0-9.0)	8.0 (7.5-8.0)	*ns*
	Pain average 7 days (0-10)	7.0 (6.0-9.0)	8.0 (7.0-9.0)	*ns*
	Pain at rest 7 days (0-10)	7.0 (6.0-8.0)	7.0 (6.5-8.0)	*ns*
	Pain Average 28 days (0-10)	7.0 (6.0-8.0)	8.0 (7.0-8.0)	*0.0308*
	Worst 28 days Pain (0-10)	9.0 (8.0-10.0)	9.0 (9.0-10.0)	*ns*
	Pain Detect (0-38)	24.0 (14.0-29.0)	24.5 (22.0-26.0)	*ns*
	SF-M Ave (0-10)	5.0 (3.0-6.7)	5.5 (4.8-6.4)	*ns*
	SF-M Neuropathic (0-10)	4.2 (2.3-7.8)	5.5 (4.3-6.5)	*ns*
	SF-M Affective (0-10)	4.1 (2.3-7.8)	5.0 (4.3-6.5)	*ns*
	SF-M Intermittent (0-10)	5.1 (1.7-7.3)	4.8 (4.0-6.3)	*ns*
	SF-M Continuous (0-10)	6.0 (4.8-7.4)	6.8 (6.2-7.4)	*0.0318*
	Temperature Sensitivity	93.3% (14/15)	81.3% (52/64)	*ns*
**Sensory**				
	PPT Left Leg (kPa)	193.0 (140.0-243.3)	239.3.0 (220.0-260.0)	*ns*
	PPT Right Arm (kPa)	150.0 (120.0-190.0)	170.0 (150.0-183.0)	*ns*
	Slow Pleasantness (-5-5)	0.0 (0.0-2.0)	0.0 (0.0-0.7)	*ns*
	Fast Pleasantness (-5-5)	0.0 (0.0-1.3)	0.0 (0.0-0.0)	*ns*

*Abbreviations: BMI, Body Mass Index; BPI, Brief Pain Inventory; EQ5D, EuroQol 5 Dimensions; FIQR, Fibromyalgia Impact Questionnaire Revised; HADS-A, Hospital Anxiety Depression Scale Anxiety; HADS-A, Hospital Anxiety Depression Scale Depression; PCS, Pain Catastrophising Scale; SF-M, Short Form McGill Pain Questionnaire; SSS, Symptom Severity Score; WPI, Widespread Pain Index. Median with (95% CI of median). Statistics: unpaired student's t test for Gaussian data, Mann Whitney U test for non-Gaussian data. Ns=not significant. * p<0.5 (uncorrected)*

Supplementary Table 2: TPE patient characteristics and qualitative outcomes

Characteristics	P1	P2	P3
**Demographics**			
Age (yrs.)	40's	20's	40's
Sex	F	F	M
Years of Diagnosis	2	3	16
**Impact indices pre TPE**			
BPI-Impact (0-10)	9.5	7.5	6.5
EQ-5D-5L Health (0-100)	40	20	45
**Impact indices post TPE**			
BPI-Impact (0-10)	4	4	2.5
EQ-5D-5L Health (0-100)	70	75	75
**Qualitative description of light touch and temperature sensitivity**			
	Patient 1 described light touch as "very sensitive and uncomfortable", associated with a feeling "like sandpaper", and they also reported that light pressure "feels like a squeeze". P1 described that standing on cold floor makes the pain "go right up their legs" and they indicated that mildly cool objects (*e.g.* a metal fork) feel painfully cold and distinguished this feeling from their pain due to touch. Ambient cold temperatures increased their spontaneous pain, but warming their body (*e.g.* a hot shower) mitigated symptoms.	Patient 2 described their pain quality as "buzzing" and indicated that low ambient temperatures (10-12°C) increased their pain. P2 also described buzzing pain after they had stroked their skin when the ambient temperature was cold.	Patient 3 described "occasional sensitivity to touch" and noted that "during a flare I also don't like to be touched". Furthermore, P3 commented that their symptoms are improved in warm and dry weather.

Qualitative description of symptom improvements after TPE

	P1 clarified that while her spontaneous pain improvement was relatively modest, sensitivity to both pressure/touch and ambient cold had dramatically improved: ‘I can take hugs without cringing or yelping’; ‘I was able to look forward to going outside rather than dreading it’ (in winter)	P2’s fatigue was reduced by 50% so that she now took on many activities around the house and felt more motivated; she also reported ‘refreshing sleep for the first time in 4 years’. Her daytime drowsiness and sleepiness had disappeared. She advised that both her fatigue and pain improvements were driving her perception of improved quality of life and function. She found activity-pacing challenging and experienced a pain flare after she had manually cleaned her own car (albeit she had performed this activity for the first time in several years).	P3 described improved sleep quality ‘I am sleeping better (6-7.5/10 vs 4.5-6/ 10 previously)’ and better control of pain flares ‘I seem to be able to tolerate more exercise and stress without flaring’. ‘I have been more physical active. Riding my e bike to the office more and longer walks with the dog.’ P3 felt ‘some reduction’ in brain fog and anxiety. Again, only modest improvement on pain were observed in the months post TPE P3 felt that their pain levels overall were ‘a bit lower’.

Supplementary Table 3: Details of microneurography subjects

Microneurography Experimental Details for Cooling Response Experiments

		Male (n = 4)		Female (n = 14)		*Total*
		HC	FMS	HC	FMS	
**Age**						
	20-24	0	0	3	0	*3*
	26-29	2	0	1	1	*3*
	30-34	2	0	1	1	*4*
	35-39	0	0	0	1	*1*
	40-44	0	0	0	1	*1*
	45-49	0	0	1	0	*1*
	50-54	0	0	1	1	*2*
	55-59	0	0	0	2	*2*
	60-64	0	0	0	0	*0*
	Total	*4*	*0*	*6*	*7*	*17*
**Nerve**						
	Radial	6	0	4	0	*10*
	S. peroneal	0	0	5	9	*14*
	Total	*6*	*0*	*8*	*9*	24
**Type**						
	SA1	3	0	4	3	*10*
	SA2	3	0	5	6	*14*
	Total	*6*	*0*	*8*	*9*	*24*

Supplementary Table 4 : Details of microneurography subjects

Microneurography Experimental Details for Mechanical Experiments

		Male (n = 5)		Female (n = 15)		*Total*
		HC	FMS	HC	FMS	
**Age**						
	20-24	1	0	3	0	*4*
	26-29	2	0	1	1	*3*
	30-34	1	0	1	1	*3*
	35-39	1	0	0	1	*2*
	40-44	0	0	0	1	*1*
	45-49	0	0	1	0	*1*
	50-54	0	0	1	1	*2*
	55-59	0	0	0	2	*2*
	60-64	0	0	0	1	*1*
	Total	*5*	*0*	*6*	*8*	*20*
**Nerve**						
	Radial	6	0	4	0	*9*
	S. peroneal	2	0	6	11	18
	Total	8	0	8	11	*29*
**Type**						
	SA1	3	0	3	3	*9*
	SA2	5	0	6	8	*18*
	Total	8	0	8	11	*29*

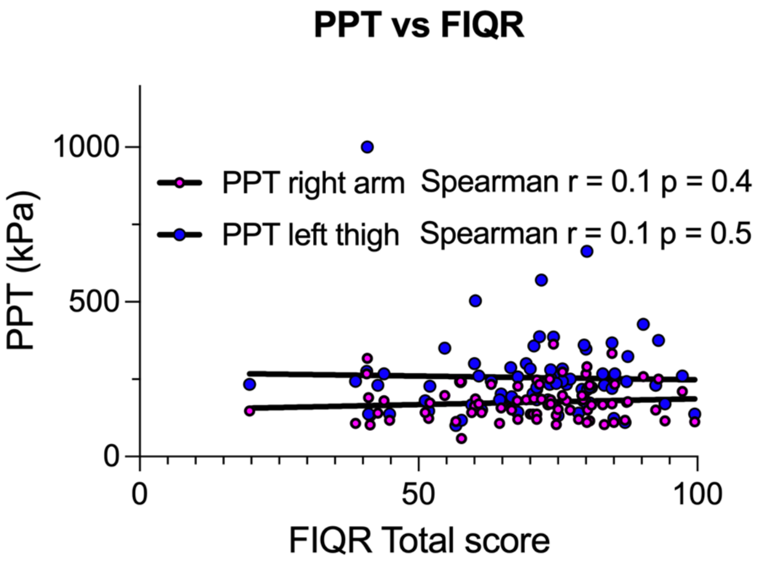

Supplementary Figure 1: Raw score pressure pain thresholds (kPa) at two sites do not correlate with impact of FM as measured by FIQR ($r = 0.1$, Spearman's correlation $p > 0.05$).

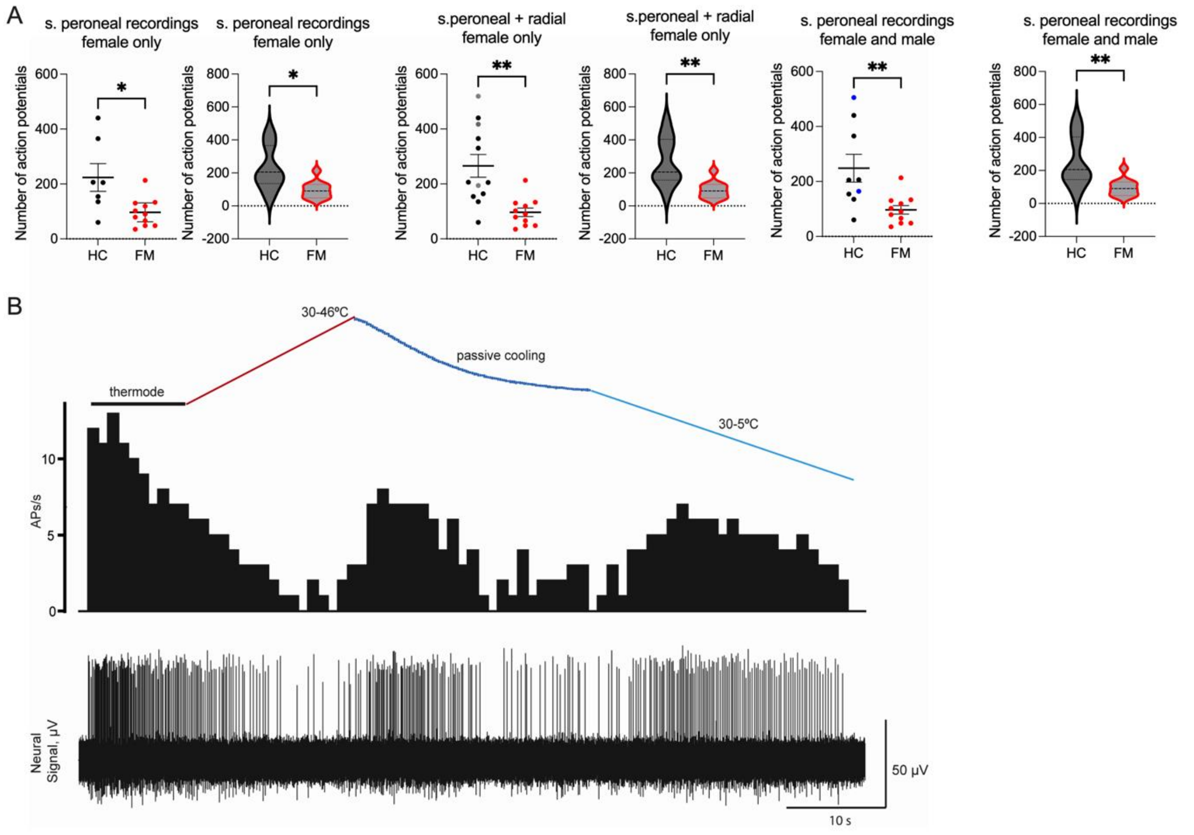

Supplementary Figure 2:
A) Breakdown of microneurography recording by sites of recording and sex of participants. There was no demonstrable difference between site and sex. Each point represents a recording. Grey dots represent female radial nerve recordings. Blue dots represent male s. peroneal nerve recordings. The same data is also presented as a violin plot to demonstrate that the spread of the data is not different between the groups. All data is presented in Figure 6.

B) Example of trace from spontaneously active (see Fig. 6G for definitions) cold sensitive AβSA unit. The unit displays high frequency firing upon the placement of the thermode which declines with time. Heating of the receptive field causes a reduction in the action potential frequency that increases upon passive cooling. Firing rate increases again during active cooling. This demonstrates that the unit is firing due to a decrease in the temperature rather than the placement of thermode on the receptive field or heating of the receptive field.
