# Supplementary material for: Aβ low threshold mechanoreceptors contribute to sensory abnormalities in fibromyalgia
Source: Brain. 2025 Sep 3;148(11):4016–29. doi: 10.1093/brain/awaf321 (PMC12588719; doi:10.1093/brain/awaf321)
Supplement: awaf321_Supplementary_Data [file awaf321_supplementary_data.zip › brain-2025-00414-File009.pdf]

Supplementary methods: Clinical Study Design and Study Subjects

In short, 'APIF' participants were identified from a registry of patients assessed for treatment with an interdisciplinary pain management program, at a tertiary national health service hospital in northern England (the Walton Centre). The consenting rate for entry into this registry is 98% of all treated patients. Patients were eligible for inclusion with FMS of over one year duration, ACR diagnosis 2010 [1] or 1990 [2] (either qualified), age above 18 years (average age: 48.9 ± 10.4, Male/Female 10/69, see Supplementary Table 1) and average weekly pain intensity ≥4/10 on a numerical rating scale. Exclusion criteria include pregnancy or breastfeeding, and concomitant autoimmune disorder (*e.g.* rheumatoid arthritis) and inadequate understanding of the English language. Following consent and confirmation of eligibility, patients were asked questions pertaining to their general health and FM symptomatology. Participants also self-completed a set of standardized questionnaires which were then checked for completeness by a member of the team. The questionnaires included the Fibromyalgia Impact Questionnaire Revised (FIQR)[3] and the McGill Short Pain Questionnaire [4]. Temperature preference was also assessed. Patients were asked if temperature affected their pain, and if so, we asked at what ambient temperature (in the range of 14 - 30°C) they had least pain. Patients were then examined for their skin sensitivity. A brief mechanical quantitative sensory testing (QST) protocol was designed to test patients' mechanical pain threshold and skin sensitivity based on the protocol previously published by Boehme *et al* [5]. Static pressure pain threshold was measured using a pressure algometer (FDN200; Wagner Instruments, Greenwich, CT, USA) with a 1 cm^2 rubber tip, which was placed on the skin and a continuous ramp of increasing intensity (approximately 0.5 kg/s, corresponding to 50 kPa/s) was applied until the patient confirmed that the sensation of pressure had changed to an additional one of pain. The patient was not able to see the dial. The pressure pain threshold was determined by the arithmetic mean of three consecutive readings. The test sites were the lateral right arm over the brachioradialis and the left leg over the vastus lateralis muscles. Participants received gentle brushstroke 10-cm in length applied manually to the skin of the lateral left forearm at slow (3 cm/s) and fast (30 cm/s) speeds from proximal to distal (with the hair growth). Subsequently, subjects rated their perceptions on a grounded 5-cm NRS for pleasantness, intensity, ticklishness, and pain - pleasantness was rated from –5 ("very unpleasant") to +5 ("very pleasant").

Participants were recruited to the DEFINE-FMS study from physiotherapy led musculoskeletal FM services, pain clinics, as well as community-based FM patient support groups. To be included in the study, patients were aged 18 years or older, satisfied the modified ACR 2016 diagnostic criteria [6] and were willing and able to provide informed consent. Exclusion criteria include other causes of neuropathy (including diabetes, prediabetes and rheumatological disorders *e.g.* Sjögren's syndrome, rheumatoid arthritis, mixed connective tissue disorders). Participants without a history of FM or any of the above exclusions were recruited as healthy volunteers (average age: 42 ± 12.5, min-max: 21 - 64, Male/Female 7/17). As part of the DEFINE-FMS protocol, individuals underwent cold pain threshold testing on the dorsum of the hand (TSA-II NeuroSensory Analyser Medoc, Ltd., Ramat-Yishai, Israel; thermode 9.0 cm^2) using ramped stimuli (30 °C – 0 °C - 1°C/sec). Individuals were asked to press the button, which terminated the ramp, immediately at the first painful sensation. This was repeated 3 times. Without verbal prompts, individuals were asked to describe the sensation at this time. A sub-group of individuals from the DEFINE-FMS study also underwent microneurography (methodology detailed below). Participants were excluded from undergoing microneurography if they were taking anti-coagulant medication or had localized skin disease in the area of testing.

We applied to the Aintree University Hospital's independent Clinical Effectiveness Steering Group (CESG) for permission to offer clinical treatment to FM patients who had tested autoantibody positive in our previously published *in vivo* study [7], and who continued to experience severe symptoms following completion of evidence-based treatments [8] including a 16-day inter-disciplinary pain management program (PMP) (See Supplementary Table 2 for details). CESG approved off-license therapeutic plasma exchange (TPE) as an experimental treatment for initially a small number of patients, recognizing that TPE is an accepted treatment-technology for patients with other immune mediated diseases, and that persuasive laboratory evidence for pertinent immune abnormalities had been presented (date of meeting: July 2018). CESG stipulated that the experimental nature of this treatment needed to be explained to patients as part of the consent procedure. Individual written informed consent was taken as per the usual procedure at the Hospital. Outcome measures were taken as per standard procedures for pain interventions at the Department of Pain Medicine. Each TPE treatment had one exchange volume over a period of 2-2.5h, and the replacement fluid was human albumin solution 4.5%. Cellular blood elements were returned. Citrate was the anticoagulant, and calcium was administered as needed if clinical

symptoms of hypocalcaemia were reported. Coagulation parameters were routinely measured, but treatment with fresh frozen plasma (FFP) was not required. We planned an extended treatment cycle of 7-8 exchange treatments (ET) over a period of 4 weeks. This protocol aimed to keep antibody levels low for a prolonged time and was based on prior experience in patients with complex regional pain syndrome (CRPS) who had typically reported slow onset of treatment effects [9]. TPE treatment for CRPS is a Category III indication in international treatment guidelines [10].

Patients completed at baseline and 2 months after treatment start (about 1 month following the last ET) a Brief Pain Inventory (BPI) short form measuring their average and worst pain intensities over the past week on an 11-point (0-10) numeric rating score with 10 = 'pain as bad as you can imagine' and the interference of their pain with activities of daily living [11], and a quality of life measure (EQ-5D-5L) [12]. These are standard periprocedural outcome measures at our Treatment Centre.

Supplementary methods: Microneurography

The inclusion criterion for this study comprised participants aged 18 years old and above. For HC participants, the exclusion criteria comprised neurological or musculoskeletal disorders, skin diseases, diabetes, and pain-relieving or psychoactive medication. FM patients were included if they had received a medical diagnosis for more than one-year. Participants were compensated for their time. The recording electrode was a 20-50mm insulated epoxy-resin tungsten recording electrode (2 and 5MΩ, 200µm diameter) was inserted percutaneously (LOGIQ P9, GE Healthcare, Chicago, IL, USA) towards either the radial or superficial peroneal nerve.

References:

1. Wolfe F, Clauw DJ, Fitzcharles MA, et al. The American College of Rheumatology preliminary diagnostic criteria for fibromyalgia and measurement of symptom severity. *Arthritis Care Res (Hoboken)*. May 2010;62(5):600-10. doi:10.1002/acr.20140

2. Wolfe F, Smythe HA, Yunus MB, et al. The American College of Rheumatology 1990 Criteria for the Classification of Fibromyalgia. Report of the Multicenter Criteria Committee. *Arthritis Rheum*. Feb 1990;33(2):160-72. doi:10.1002/art.1780330203

3. Bennett RM, Friend R, Jones KD, Ward R, Han BK, Ross RL. The Revised Fibromyalgia Impact Questionnaire (FIQR): validation and psychometric properties. *Arthritis Res Ther*. 2009;11(4):R120. doi:10.1186/ar2783

4. Dworkin RH, Turk DC, Revicki DA, et al. Development and initial validation of an expanded and revised version of the Short-form McGill Pain Questionnaire (SF-MPQ-2). *Pain*. Jul 2009;144(1-2):35-42. doi:10.1016/j.pain.2009.02.007

5. Boehme R, Van Ettinger-Veenstra H, Olausson H, Gerdle B, Nagi S. Anhedonia to Gentle Touch in Fibromyalgia: Normal Sensory Processing but Abnormal Evaluation. *Brain Sciences*. 05/18 2020;10doi:10.3390/brainsci10050306

6. Wolfe F, Clauw DJ, Fitzcharles MA, et al. 2016 Revisions to the 2010/2011 fibromyalgia diagnostic criteria. *Semin Arthritis Rheum*. Dec 2016;46(3):319-329. doi:10.1016/j.semarthrit.2016.08.012

7. Goebel A, Krock E, Gentry C, et al. Passive transfer of fibromyalgia symptoms from patients to mice. *J Clin Invest*. Jul 1 2021;131(13)doi:10.1172/jci144201

8. Macfarlane GJ, Kronisch C, Dean LE, et al. EULAR revised recommendations for the management of fibromyalgia. *Ann Rheum Dis*. Feb 2017;76(2):318-328. doi:10.1136/annrheumdis-2016-209724

9. Goebel A, Jones S, Oomman S, Callaghan T, Sprotte G. Treatment of Long-Standing Complex Regional Pain Syndrome with Therapeutic Plasma Exchange: A Preliminary Case Series of Patients Treated in 2008–2014. *Pain Medicine*. 2014;15(12):2163-2164. doi:10.1111/pme.12601

10. Schwartz J, Padmanabhan A, Aqui N, et al. Guidelines on the Use of Therapeutic Apheresis in Clinical Practice-Evidence-Based Approach from the Writing Committee of the American Society for Apheresis: The Seventh Special Issue. *J Clin Apher*. Jun 2016;31(3):149-62. doi:10.1002/jca.21470

11. Tan G, Jensen MP, Thornby JI, Shanti BF. Validation of the Brief Pain Inventory for chronic nonmalignant pain. *J Pain*. Mar 2004;5(2):133-7. doi:10.1016/j.jpain.2003.12.005

12. Herdman M, Gudex C, Lloyd A, et al. Development and preliminary testing of the new five-level version of EQ-5D (EQ-5D-5L). *Qual Life Res*. Dec 2011;20(10):1727-36. doi:10.1007/s11136-011-9903-x
