# Supplementary material for: Aβ low threshold mechanoreceptors contribute to sensory abnormalities in fibromyalgia
Source: Brain. 2025 Sep 3;148(11):4016–29. doi: 10.1093/brain/awaf321 (PMC12588719; doi:10.1093/brain/awaf321)
Supplement: awaf321_Supplementary_Data [file awaf321_supplementary_data.zip › brain-2025-00414-File010.pdf]

Author contributions:

MRI conceived, designed, performed experiments including electrophysiology and animal behavior and analyzed data including clinical, electrophysiology, *in vivo* calcium imaging and microneurography, and wrote and edited the manuscript. RB designed, conducted and collated the APIF clinical experiments and contributed to writing and editing the manuscript. NV performed electrophysiology experiment and contributed to writing the manuscript. QZ performed the *in vivo* calcium imaging experiment. MM analyzed experimental data and contributed to the editing of the manuscript. HS assisted with animal behavioral studies. KP assisted with data collection for APIF clinical experiments. WM assisted with data collection and data collation for microneurography experiments. JD performed microneurography experiments in HV and individuals with FMS. HN performed sample IgG preparation. CG designed and performed behavioral experiments. Anne M collected clinical and behavioral data for the DEFINE-FMS study, performed microneurography experiments in HV and individuals with FMS, contributed to the writing of the manuscript. OB, SN and HO collected microneurography data in HV. UA was the PI on DEFINE-FMS study. XD conceived of and designed experiments. Andrew M designed and performed microneurography recordings, collected microneurography data in HV and individuals with FMS, contributed to the writing and editing of the manuscript. SB contributed to experimental design and edited the manuscript. AG, conceived and initiated the clinical studies including therapeutic plasma exchange, designed and conducted clinical aspects, and contributed to experimental design and to writing and editing the manuscript. DAA conceived of and designed experiments and analysis, contributed to the writing and editing of the manuscript. All authors participated in the critical reading of the manuscript and gave their consent for the final draft.
